# Association of Extracellular Signal-Regulated Kinase Genes With Myopia: A Longitudinal Study of Chinese Children

**DOI:** 10.3389/fgene.2021.654869

**Published:** 2021-05-27

**Authors:** Haishao Xiao, Shudan Lin, Dandan Jiang, Yaoyao Lin, Linjie Liu, Qiqi Zhang, Juan He, Yanyan Chen

**Affiliations:** ^1^School of Optometry and Ophthalmology, Wenzhou Medical University, Wenzhou, China; ^2^The Eye Hospital, Wenzhou Medical University, Wenzhou, China

**Keywords:** single nucleotide polymorphism, biological networks, association analysis, schoolchildren myopia, ocular parameters

## Abstract

**Objective:**

The present study was designed to investigate whether the extracellular signal-regulated kinase (ERK) signaling pathway, a downstream component of dopamine signaling, is involved in myopia among Chinese children.

**Methods:**

During a 3.5-year follow-up, 488 primary school students were enrolled in this study. Non-cycloplegic spherical equivalent refraction (SE) and other ocular parameters were assessed. Four variants of four genes in the ERK signaling pathway were selected: *RASGRF1* rs6495367, *PTPN5* rs1550870, *PTPRR* rs11178469, and *PDGFRA* rs6554163. SNPscan was used to genotype single-nucleotide polymorphisms (SNPs). PLINK software was used to assess the associations of the genetic variants with the occurrence or development of myopia, SE, and other ocular parameters. We created a protein-protein interaction (PPI) network and microRNA (miRNA)-gene network using String and Cytoscape and conducted enrichment analyses on the genes in these networks.

**Results:**

In total, 426 children (baseline age: 7.28 ± 0.26 years; 236 (55.4%) boys and 190 girls) wereenrolled. After adjusting for confounding factors with 10,000 permutations, children with the CT or TT genotype of *PTPN5* rs155087*0* were more susceptible to myopia than those with the CC genotype (adjusted *p* = 0.011). Additionally, *PTPN5* rs155087*0* was correlated with significant myopic shift and increasing axial length (AL) and lens thickness (LT) but had a negative effect on central corneal thickness (CCT). *RASGRF1* rs6495367 was negatively associated with myopic shift (additive: adjusted *p* = 0.034; dominant: adjusted *p* = 0.020), myopic SE and AL. *PDGFRA* rs6554163 TA or AA was negatively associated with increasing LT (adjusted *p* = 0.033). Evaluation of the effects of SNP-SNP combinations on incident myopia revealed a statistically significant one-locus model: *PTPN5* rs1550870 [cross-validation consistency (CVC) = 10/10, adjusted *p* = 0.0107]. The genes in the PPI and miRNA-gene interaction networks were subjected to enrichment analyses, which suggested that these genes are involved mainly in eye development and dopaminergic synapse-related processes.

**Conclusion:**

We identified genetic variants of crucial ERK signaling pathway genes that were significantly correlated with myopia and ocular parameter alterations in Chinese children. A combination of gene and miRNA functional analyses with enrichment analyses highlights the regulatory effects associated with ocular development and dopamine biological functions. This study offers novel clues to understand the role of dopamine in the molecular mechanisms of myopia.

## Graphical Abstract

The genes in the miRNA-target gene network represent the intersection of the target genes and the genes from String that had direct or indirect interaction relationships with significant genes.

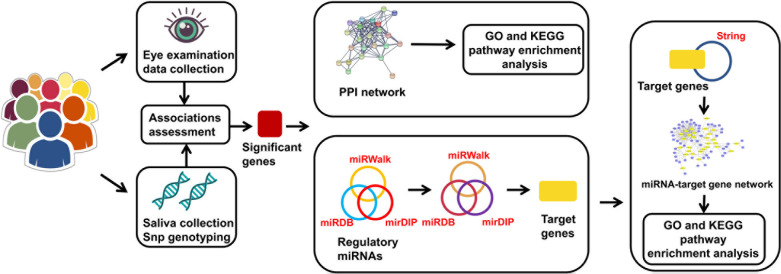

## Introduction

Myopia is a common but vision-threatening disorder for humans worldwide, especially those of Asian ancestry. Pediatric myopia, the prevalence of which has increased in recent years, has emerged as a major public concern (Cho and Tan, 2019). A study by Wang et al. (2018) revealed that Chinese students have a higher incidence of myopia than individuals of any other cultural or ethnic group. Environmental factors can significantly contribute to myopia (Pan et al., 2012), and epidemiological studies have reported multiple risk factors, including near-work activities and a lack of outdoor activities (Sun et al., 2018; Huang et al., 2020). In addition, myopia is highly heritable. According to a twin study, the estimated heritability of myopia is up to 90% (Hammond et al., 2001). Moreover, genome-wide association studies (GWASs), which have demonstrated remarkable progress in dissecting the genetic backgrounds of disease in recent years, have revealed hundreds of genetic variants and polymorphisms associated with myopia and refractive error (Tedja et al., 2019; Hysi et al., 2020). However, the specific mechanisms underlying myopia remain unclear.

Dopamine (DA), an important neurotransmitter, has been confirmed to exist in the retina and to mediate diverse functions including visual signaling and refractive development. In recent years, numerous studies have tested the hypothesis that the release of DA in the retinas can control myopia (Stone et al., 1989; Zhou et al., 2017). In addition, the mechanisms by which outdoor activity and bright light exposure inhibit myopia are likely to be mediated by DA (Ashby et al., 2009; Chen et al., 2017; Zhou et al., 2017). Although the key roles of DA and its receptors, such as D1-like (D1) receptors and D2-like (D2) receptors, in modulating visual function and refractive development have been verified, the exact downstream components that transduce DA activation signals in the retina to control myopia are largely undefined (Zhou et al., 2017).

An analysis of mouse retinas showed that retinal ganglion cells (RGCs) express both D1 and D2 receptors. The mitogen-activated protein kinase (MAPK)/extracellular signal-regulated kinase (ERK) signaling pathway may also be involved in modulation of neuronal functions mediated by the D1 receptor (Li et al., 2016). Additionally, DA activates the ERK signaling pathway via D2 receptors (Welsh et al., 1998). A previous study indicated that as a downstream component in the DA signaling pathway, the ERK signaling pathway assembles multiple effects of transduction cascades coupled with D2 receptors that are expressed in chicken photoreceptors (Ko et al., 2003). Given all this evidence, we hypothesized that the ERK signaling pathway is one of the downstream signaling cascades of DA in the retina that controls myopia.

As a member of the MAPK family, ERK aids in the transmission of extracellular signals to intracellular proteins, thus playing key roles in cell proliferation, differentiation, migration, senescence and apoptosis (Abe et al., 2002; Sun et al., 2015).

In the ERK pathway, platelet-derived growth factor (PDGF) receptor alpha (PDGFRA) is a member of the receptor tyrosine kinase family and relies on ERK to promote cell viability (Hayashi et al., 2015). A GWAS of an Asian population from Singapore showed that a single-nucleotide polymorphism (SNP) of the *PDGFRA* gene was associated with corneal curvature, and this finding was subsequently verified in Australian and European populations (Han et al., 2011; Mishra et al., 2012; Guggenheim et al., 2013). RAS protein-specific guanine nucleotide releasing factor 1 (*RASGRF1*) phosphorylates members of the ERK pathway to regulate downstream cellular signaling molecules (Tsai et al., 2018). *RASGRF1* was revealed to be related to refractive error in previous GWASs involving Asian and European participants (Hysi et al., 2010; Tedja et al., 2018). In addition, previous studies have demonstrated its high expression in the retina and have identified it as a strong candidate gene for association with high myopia (Chen et al., 2015). Protein tyrosine phosphatase non-receptor type 5 (*PTPN5*) is expressed in brain regions related to adult neuroplasticity. It is able to inactivate ERK1/2 and restrict the distribution of ERK signaling (Olausson et al., 2012). *PTPN5* rs1550870 has been found to be strongly associated with myopia in a large-sample GWAS based on a European population (Pickrell et al., 2016). As an important paralog of *PTPN5*, *PTPRR* is a key negative regulator of the ERK signaling pathway (Shi et al., 2012). In a Caucasian family cohort study, *PTPRR* rs3803036 was found to be strongly associated with high myopia (Hawthorne et al., 2013).

According to previous studies, each of the four genes described above that plays a pivotal role in the ERK signaling pathway is associated with myopia to varying degrees. However, to the best of our knowledge, few studies have been carried out thus far to investigate the ERK pathway in its entirety and to explore its comprehensive involvement in the onset and development of myopia. In addition, ocular traits are closely related to the refractive status of the eye. Based on the information provided above, we conducted a longitudinal study to collect data on the refractive status and ocular parameters in primary school children. We assessed DNA in saliva from these children and performed bioinformatic analyses to further investigate whether the polymorphisms of these four genes in the ERK signaling pathway are strongly associated with myopia and thus to illuminate the role of the ERK pathway in DA-mediated myopia control.

## Materials and Methods

### Study Subjects and Phenotype Assessment

The subjects involved in this 3.5-year prospective longitudinal study were recruited from among second grade students at primary schools in the Lucheng District of Wenzhou, Zhejiang, China, from September 2014 to May 2018. Three schools were selected using stratified random sampling according to the similar socioeconomic statuses and educational backgrounds of the students and to their similar resources. A total of 487 students were recruited, but we excluded some students who met the following criteria: (1) wore orthokeratology lenses, (2) had serious eye diseases that may affect refraction, (3) were undergoing ocular surgery, or (4) were lost to follow-up or had incomplete information. All participants underwent comprehensive ophthalmologic examinations including measurement of the non-cycloplegic spherical equivalent (SE, RM-8900; Topcon Corp, Tokyo, Japan), axial length (AL), corneal radius of curvature (CRC), anterior chamber depth (ACD), central corneal thickness (CCT), and lens thickness (LT) (IOL Master; Carl Zeiss Meditec, Oberkochen, Germany). In addition, the participants were asked to complete a questionnaire to collect detailed information on the short-distance use of eyes and the time spent outdoors. Myopia was defined as an SE ≤ −1.0 diopter (D) (Rim et al., 2017), and if a child’s average shift in myopic SE was ≥ 0.5 D per year (Wei et al., 2020) during the follow-up period, we considered the child to exhibit significant myopic progression. Additionally, incident myopia was defined as an absence of myopia at baseline but the development of myopia during the follow-up period. As there were no significant differences in refractive data between the left and right eyes (Spearman’s ρ = 0.86–0.91), only the data for the right eye were analyzed.

All procedures in this study were performed following the tenets of the Declaration of Helsinki. The study protocol was approved by the Ethics Committee of the Eye Hospital of Wenzhou Medical University [No. KYK (2014)3]. All the participants and their guardians were fully informed of the purpose and procedures, and written consent was obtained from each participant.

### Selection and Genotyping of SNPs

To select SNPs for these four genes, before a literature review, candidate SNPs were selected from 2 databases (SNPedia, the GWAS Catalog) and one study (the CREAM Consortium study). Information on these 4 genes is shown in Table 1. Genomic DNA was extracted from the buccal swab specimen of each participant. Standard procedures were followed. First, variant genotyping was performed by double ligation and multiplex fluorescence PCR using a custom-designed 48-Plex SNPscan^TM^ Kit (Cat#: G0104; Genesky Biotechnologies, Inc., Shanghai, China). Second, DNA denaturing was conducted in an ABI2720 thermal cycler. Then, the resulting product was mixed with a 10-mL ligation premix. Each ligation product required two 48-plex fluorescence PCR runs. An ABI3730XL sequencer was chosen to perform capillary electrophoresis for PCR product separation and detection. Information on the labeling dye color and fragment size of each allele-specific ligation-PCR product was collected to analyze the raw data. In addition, to ensure high quality and repeatability, 3% duplicate samples were tested to confirm the genotyping results.

**TABLE 1:** Primary information on the *PDGFRA* rs6554163 T > A, *RASGRF1* rs6495367 G > A, *PTPN5* rs1550870 C > T, *PTPRR* rs11178469 T > C polymorphisms.

				Control group vs. incident myopia group		Control group vs. significant myopic shift group		
Gene	SNP	MAF^*a*^ for the Chinese population^*b*^	Call rate (%)	MAF^*a*^ in our controls (*n* = 174)	*P*-value for the HWE^*c*^ test in our controls	MAF^*a*^ in our controls (*n* = 281)	*P*-value for the HWE^*c*^ test in our controls	*P*-value in GWAS
*PDGFRA*	rs6554163 T > A	0.221	99.10	0.21	0.259	0.20	0.266	2.8 × 10^–6^ (Guggenheim et al., 2013)
*RASGRF1*	rs6495367 G > A	0.490	98.58	0.49	0.546	0.48	0.340	1.95 × 10^–24^ (Tedja et al., 2018)
*PTPN5*	rs1550870 C > T	0.296	98.82	0.27	0.570	0.27	0.176	9.9 × 10^–13^ (Pickrell et al., 2016)
*PTPRR*	rs11178469 T > C	0.394	99.13	0.41	1	0.41	0.622	1.33 × 10^–13^ (Tedja et al., 2018)

*^*a*^Minor allele frequency. ^*b*^Refer to https://www.internationalgenome.org/category/dbsnp. ^*c*^Hardy-Weinberg equilibrium.*

### Functional Annotation

We used HaploReg v 4.1 and RegulomeDB to functionally annotate these four genes. HaploReg has emerged as an important tool for the annotation of variants in haplotype blocks in the non-coding genome and for the prediction of cell types that are likely affected (Ward and Kellis, 2012; Yu et al., 2019). RegulomeDB can be used to annotate regulatory variants in the human genome by giving scores to predict their functions (Boyle et al., 2012).

### Protein-Protein Interaction Network

String^1^ was used to create protein-protein interaction (PPI) networks and perform pathway enrichment analysis on the significant genes (Szklarczyk et al., 2019). The selected settings were as follows: minimum required interaction score, highest confidence (0.900); first shell, no more than 20 interactors; and second shell, none. The rest of the settings were the default settings.

### MicroRNA-Gene Interaction Network

MicroRNAs involved in the regulation of the genes with significant results in the statistical analyses were predicted using miRWalk 2.0^2^, miRDB^3^, and mirDIP^4^. MiRWalk contains complete sequence information, including information on 5′-UTRs, CDSs and 3′-UTRs (Sticht et al., 2018). To reduce the occurrence of false-positive results, the screening standards were set as follows: miRWalk, score >0.8; miRDB, score >80; and mirDIP score class, very high. Additionally, a Venn diagram^5^ was employed to reveal the miRNAs that existed in all three databases. Finally, all the miRNAs were merged to target the relevant genes by utilizing the above databases with the same standards.

To thoroughly investigate the functional regulation between miRNAs and genes, we focused on the miRNAs that had direct or indirect interactions with our significant genes. String was used to select the important genes from the abundant target genes with specific settings, including medium confidence (0.4) and no more than 50 interactors for both the first and second shells. A miRNA-gene network was then visualized with Cytoscape v3.8.2^6^, a software program that enables the integration, visualization and analysis of molecular interaction networks (Shannon et al., 2003).

### GO and KEGG Enrichment Analyses

Each set of genes utilized in the construction of the PPI and miRNA-gene interaction networks was subjected to Gene Ontology (GO) and Kyoto Encyclopedia of Genes and Genomes (KEGG) enrichment analyses with a false discovery rate (FDR) less than 0.05. Both of these databases are available on the String website. GO is a comprehensive database that can be used to annotate genes, gene products, and sequences. KEGG is widely used for the biological interpretation of genomic sequences.

### Data Analysis

In this study, we used SPSS version 25.0 (IBM, Armonk, NY, United States) for descriptive statistics and PLINK 1.9 for regression analyses. The Hardy-Weinberg equilibrium (HWE) test was performed for all SNPs using PLINK, and a *p* > 0.05 suggested that the SNP occurrence was consistent with HWE. Logistic regression models were used to investigate the allelic associations of each SNP with the occurrence and development of myopia, with odds ratios (ORs) and 95% confidence intervals (CIs) as the measurement indexes. In addition, associations of SNPs and ocular traits were evaluated by linear regression analysis. All the regression analyses were adjusted for the confounding factors of age, sex, near-work time, outdoor time, and corresponding baseline traits related to the outcome indicators. Significance was set at *p* < 0.05, and we employed 10,000 permutations for multiple comparisons using the max(T) permutation procedure in PLINK. In addition, general multifactor dimensionality reduction (GMDR) was applied to investigate gene-gene interactions. In various population-based researches, this method permits adjustment for quantitative covariates and is applicable to continuous and dichotomous phenotypes; in accordance with the degree of consistency, GMDR software provides the cross-validation consistency (CVC) score when a selected interaction is identified as the best model among all possibilities considered (Lou et al., 2007). The testing balanced accuracy provides the scores between 0.50 (no better than chance) and 1.00 (perfect prediction) to measure the degree of interaction that predicts the case-control status. When the score is higher than 0.5 out of 10 cross-validation cases, the sign test counts the number of cases and the corresponding *p*-value indicates the probability of getting these cases of prediction accuracy higher than 0.5 out of ten cases with random prediction (Kim et al., 2008). The best model is selected as the combination of SNPs with the maximum CVC score, the best prediction accuracy, and a significant *p*-value (Aung et al., 2014).

## Results

### Characteristics of the Study Population

After excluding 29 students who wore orthokeratology lenses, one student with glaucoma, one student with amblyopia and 32 students with incomplete genetic or ocular examination information, a total of 426 participants were ultimately included in this analysis. Of the included participants, 55.4% were male, and the average age was 7.28 ± 0.26 years. Forty-nine children were myopic at baseline and were excluded from the logistic analysis of incident myopia. Table 2 shows the quantitative traits and demographic information on the participants.

**TABLE 2:** Characteristics of the participants.

Variable	
Total number	426
Age (years)	7.28 ± 0.46
Males, N (%)	236 (55.4%)
Baseline Myopia, N (%)	49 (11.5)
Baseline SE (D)^*a*^	0.00 (−0.50, 0.33)
Baseline AL (mm)^*b*^	22.97 ± 0.76
Baseline CRC (mm)^*b*^	7.80 ± 0.26
Baseline CCT (mm)^*b*^	0.54 ± 0.03
Baseline LT (mm)^*b*^	3.57 ± 0.18
Baseline ACD (mm)^*b*^	2.92 ± 0.25
ΔSE (D/y)^*a*^	−0.32 (−0.60, −0.10)
ΔSE > −0.50 (D/y), N (%)	145 (34.0%)
ΔAL (mm/y)^*a*^	0.30 (0.20, 0.40)
ΔCRC (mm/y)^*a*^	−0.016 (−0.03, −0.01)
ΔCCT (mm/y)^*a*^	2.00 (0.95, 3.14)
ΔLT (mm/y)^*a*^	−0.04 (−0.08, 0.01)
ΔACD (mm/y)^*a*^	0.05 (0.04, 0.07)

*SE, spherical equivalent refraction; D, diopter; AL, axial length; CRC, corneal radius of curvature; CCT, central corneal thickness; ACD, anterior chamber depth. Δ is the change in the SE, AL, CRC, CCT, LT, and ACD per year during the 3.5-year follow-up. ^*a*^Median (Q1, Q3); ^*b*^mean ± SD.*

Additionally, Table 1 summarizes the gene symbols, minor allele frequencies (MAFs), HWE values, and call rates for all SNPs. The SNP distributions in the controls of both subgroups were consistent with HWE.

### Functional Evaluation of Selected SNPs

The HaploReg v4.1 prediction revealed that three SNPs (*PDGFRA* rs6554163, *RASGRF1* rs6495367, and *PTPN5* rs155087*0*) were regulatory SNPs. Specifically, *PDGFRA* rs6554163 was predicted to be located within promoter histone marks of 4 tissues, enhancer histone marks of 5 tissues, and a DNase hypersensitivity region and to significantly alter 3 motifs (BAF155, SIX5, and Znf143). In addition, *PTPN5* rs155087*0* was predicted to be located in enhancer histone marks and to alter 5 motifs. The *RASGRF1* rs6495367 variant was predicted to change the SIX5 motif. The ranks of *PDGFRA* rs6554163, *PTPN5* rs155087*0*, and *PTPRR* rs11178469 provided by RegulomeDB were 5, suggesting transcription factor (TF) binding or DNase peaks for these 3 SNPs. *RASGRF1* rs6495367 is likely to exhibit both TF binding and a DNase peak, as its rank was 4. More details are given in Table 3.

**TABLE 3:** Genetic information and functional annotation of 4 SNPs using HaploReg v4.1 and RegulomeDB.

						HaploReg v4.1				
SNP	Gene	Chr	Position^a^	Allele	Functional consequence	Promoter histone marks	Enhancer histone marks	DNAse	Motifs changed	RegulomeDB Rank
rs6554163	*PDGFRA*	4	55102559	T > A	intron	4 tissues	5 tissues	ESDR	BAF155, SIX5, Znf143	5^c^
rs6495367	*RASGRF1*	15	79375347	G > A	intron	NA^b^	NA	NA	SIX5	4^d^
rs1550870	*PTPN5*	11	18751041	C > T	synonymous	NA	IPSC, ESC, SKIN	NA	CACD_1, CACD_2, Klf4, Klf7, PU.1_disc3, Spz1_1	5
rs11178469	*PTPRR*	12	71275137	T > C	intron	NA	NA	NA	NA	5

*^*a*^Genomic position information is based on Genome Reference Consortium Human Build 37 (GRCh37). ^*b*^NA Not available. ^*c*^5: TF binding or DNase peak. ^*d*^4: TF binding + DNase peak.*

### Associations Between Selected SNPs and Incident Myopia or Significant Myopic Shift

After adjusting for confounding variables, such as age, sex, near-work time, outdoor time, and baseline SE, children with the CT or TT genotype of *PTPN5* rs155087*0* were found to be more susceptible to myopia than those with the CC genotype (dominant: OR = 1.885; 95% CI = 1.152–3.086, adjusted *p* = 0.011, Table 4). Apart from this, no statistically significant relationships with incident myopia were observed for other SNPs. Details regarding the correlations among the SNPs and incident myopia are presented in Table 4.

**TABLE 4:** Distribution of genotypes and alleles of SNPs in the control and incident myopia groups.

		Incident myopia group (*n* = 202)		Control group (*n* = 174)				
SNP	Genotype	No.	%	No.	%	OR (95% CI)^*c*^	*P*^*a*^	*P*^*b*^
***PDGFRA*** rs6554163 **T > A**								
Additive	AA/TA/TT	−	−	−	−	0.713 (0.462, 1.099)	0.125	0.124
Dominant	TT	135	66.8	105	60.3	Ref.		
	TA + AA	67	33.2	69	39.7	0.639 (0.390, 1.046)	0.075	0.073
Recessive	TA + TT	196	97.0	169	97.1	Ref.		
	AA	6	3.0	5	2.9	1.046 (0.280, 3.906)	0.947	0.959
***RASGRF1*** rs6495367 **G > A**								
Additive	AA/GA/GG	−	−	−	−	0.959 (0.685, 1.344)	0.809	0.808
Dominant	GG	44	21.8	47	27.0	Ref.		
	GA + AA	158	78.2	127	73.0	0.832 (0.472, 1.467)	0.526	0.533
Recessive	GA + GG	147	72.8	130	74.7	Ref.		
	AA	55	27.2	44	25.3	1.060 (0.622, 1.807)	0.830	0.836
***PTPN5*** rs1550870 **C > T**								
Additive	TT/CT/CC	−	−	−	−	1.454 (0.961, 2.199)	0.076	0.077
Dominant	CC	83	41.1	91	52.3	Ref.		
	CT + TT	119	58.9	83	47.7	1.885 (1.152, 3.086)	**0.012**	**0.011**
Recessive	CT + CC	191	94.6	163	93.7	Ref.		
	TT	11	5.4	11	6.3	0.634 (0.229, 1.753)	0.380	0.371
***PTPRR*** rs11178469 **T > C**								
Additive	CC/TC/TT	−	−	−	−	0.993 (0.699, 1.410)	0.969	0.970
Dominant	TT	71	35.1	61	35.1	Ref.		
	TC + CC	131	64.9	113	64.9	1.106 (0.672, 1.821)	0.692	0.692
Recessive	TC + TT	173	85.6	145	83.3	Ref.		
	CC	29	14.4	29	16.7	0.817 (0.421, 1.583)	0.549	0.551

*^*a*^Adjusted for age, sex, near-work time, outdoor time, and baseline SE. ^*b*^Adjusted for age, sex, near-work time, outdoor time, and baseline SE with 10,000 permutations. ^*c*^CI, confidence interval. The bold values are P < 0.05.*

With regard to the associations of significant myopic shift with all SNPs, *RASGRF1* rs6495367 was negatively associated with myopic shift (additive: adjusted *p* = 0.034; dominant: adjusted *p* = 0.020). In addition, *PTPN5* rs155087*0* was found to be correlated with significant myopic shift in both the additive and dominant models (additive: adjusted *p* = 0.025; dominant: adjusted *p* = 0.016) (Table 5).

**TABLE 5:** Distribution of genotypes and alleles of SNPs in the control and significant myopic shift groups.

		Significant myopic shift group (*n* = 202)		Control group (*n* = 174)				
SNP	Genotype	No.	%	No.	%	OR (95% CI)^c^	*P*^a^	*P*^b^
***PDGFRA*** rs6554163 **T > A**								
Additive	AA/TA/TT	−	−	−	−	0.776 (0.513, 1.174)	0.230	0.232
Dominant	TT	97	66.9	176	62.6	Ref.		
	TA + AA	48	33.1	105	37.4	0.748 (0.470, 1.189)	0.219	0.223
Recessive	TA + TT	141	97.2	273	97.2	Ref.		
	AA	4	2.8	8	2.8	0.771 (0.198, 3.003)	0.707	0.712
***RASGRF1*** rs6495367 **G > A**								
Additive	AA/GA/GG	−	−	−	−	0.710 (0.519, 0.973)	**0.033**	**0.034**
Dominant	GG	22	15.2	132	47.0	Ref.		
	GA + AA	123	84.8	149	53	0.510 (0.288, 0.903)	**0.021**	**0.020**
Recessive	GA + GG	102	70.3	211	75.1	Ref.		
	AA	43	29.7	70	24.9	0.744 (0.459, 1.206)	0.230	0.236
***PTPN5*** rs1550870 **C > T**								
Additive	TT/CT/CC	−	−	−	−	1.515 (1.050, 2.186)	**0.027**	**0.025**
Dominant	CC	58	40	144	51.2	Ref.		
	CT + TT	87	60	137	48.8	1.774 (1.119, 2.811)	**0.015**	**0.016**
Recessive	CT + CC	133	91.7	265	94.3	Ref.		
	TT	12	8.3	16	5.7	1.288 (0.549, 3.025)	0.561	0.561
***PTPRR*** rs11178469 **T > C**								
Additive	CC/TC/TT	−	−	−	−	0.987 (0.710, 1.374)	0.940	0.940
Dominant	TT	49	33.8	97	34.5	Ref.		
	TC + CC	96	66.2	184	65.5	1.026 (0.642, 1.639)	0.916	0.914
Recessive	TC + TT	124	85.5	237	84.3	Ref.		
	CC	21	14.5	44	15.7	0.914 (0.487, 1.712)	0.778	0.780

*^*a*^Adjusted for age, sex, near-work time, outdoor time, and baseline SE. ^*b*^Adjusted for age, sex, near-work time, outdoor time, and baseline SE with 10,000 permutations. ^*c*^CI, confidence interval. The bold values are P < 0.05.*

### Associations Between Selected SNPs and Quantitative Traits

Based on the linear regression results shown in Table 6, as a protective factor, *RASGRF1* rs6495367 was significantly related to myopic SE (additive: coefficient = 0.061, adjusted *p* = 0.037; dominant: coefficient = 0.114, adjusted *p* = 0.019) and AL (dominant: coefficient = −0.035, adjusted *p* = 0.047). In addition, *PTPN5* rs155087*0* appeared to be associated with increased AL (dominant: coefficient = 0.033, adjusted *p* = 0.025) and LT (additive: coefficient = 0.040, adjusted *p* = 0.041), while it had a negative effect on CCT (additive: coefficient = −0.350, adjusted *p* = 0.033; dominant: coefficient = −0.460, adjusted *p* = 0.019). The analysis also revealed a statistically significant association of *PDGFRA* rs6554163 with increased LT, but the association existed only in the dominant model (coefficient = −0.053, adjusted *p* = 0.033).

**TABLE 6:** Associations of SNPs with quantitative ocular traits in different genetic models.

		Additive			Dominant			Recessive		
Variable	SNP	β^*a*^ (95% CI)	*P*^*a*^	*P*^*b*^	β^*a*^ (95% CI)	*P*^*a*^	*P*^*b*^	β^*a*^ (95% CI)	*P*^*a*^	*P*^*b*^
**Δ SE**, D/y	*PDGFRA* rs6554163	0.018 (−0.056, 0.091)	0.638	0.634	0.022 (−0.062, 0.106)	0.607	0.606	0.007 (−0.229, 0.243)	0.956	0.958
	*RASGRF1* rs6495367	0.061 (0.004, 0.117)	**0.036**	**0.037**	0.114 (0.019, 0.210)	**0.019**	**0.019**	0.051 (−0.039, 0.141)	0.266	0.265
	*PTPN5* rs155087*0*	−0.055 (−0.122, 0.012)	0.107	0.117	−0.080 (−0.162, 0.002)	0.056	0.055	−0.011 (−0.174, 0.152)	0.898	0.896
	*PTPRR* rs11178469	0.009 (−0.052, 0.069)	0.779	0.774	−0.008 (−0.094, 0.078)	0.856	0.857	0.044 (−0.069, 0.157)	0.444	0.439
**Δ AL**, mm/y	*PDGFRA* rs6554163	0.012 (−0.015, 0.038)	0.394	0.402	0.010 (−0.020, 0.040)	0.524	0.523	0.042 (−0.045, 0.129)	0.349	0.351
	*RASGRF1* rs6495367	−0.018 (−0.038, 0.003)	0.092	0.089	−0.035 (−0.069, −0.001)	**0.046**	**0.047**	−0.013 (−0.045, 0.019)	0.430	0.436
	*PTPN5* rs155087*0*	0.023 (−0.001, 0.047)	0.060	0.056	0.033 (0.004, 0.063)	**0.025**	**0.025**	0.004 (−0.053, 0.062)	0.878	0.880
	*PTPRR* rs11178469	−0.007 (−0.029, 0.014)	0.520	0.522	0.003 (−0.028, 0.033)	0.861	0.862	−0.030 (−0.070, 0.011)	0.149	0.148
**Δ CRC**, mm/y	*PDGFRA* rs6554163	−0.002 (−0.005, 0.002)	0.350	0.351	−0.002 (−0.005, 0.002)	0.360	0.362	−0.002 (−0.013, 0.009)	0.694	0.695
	*RASGRF1* rs6495367	0.000 (−0.002, 0.003)	0.739	0.733	0.000 (−0.005, 0.004)	0.875	0.875	0.001 (−0.003, 0.005)	0.498	0.507
	*PTPN5* rs155087*0*	0.001 (−0.002, 0.004)	0.585	0.578	0.000 (−0.004, 0.004)	0.998	0.997	0.005 (−0.002, 0.012)	0.188	0.184
	*PTPRR* rs11178469	−0.001 (−0.004, 0.001)	0.314	0.321	−0.002 (−0.006, 0.002)	0.339	0.340	−0.002 (−0.007, 0.003)	0.533	0.529
**Δ CCT**, mm/y	*PDGFRA* rs6554163	0.233 (−0.118, 0.584)	0.194	0.196	0.254 (−0.144, 0.652)	0.211	0.216	0.360 (−0.762, 1.481)	0.530	0.502
	*RASGRF1* rs6495367	0.042 (−0.230, 0.313)	0.763	0.767	0.142 (−0.318, 0.602)	0.546	0.549	−0.019 (−0.448, 0.410)	0.931	0.933
	*PTPN5* rs155087*0*	−0.350 (−0.668, −0.032)	**0.032**	**0.033**	−0.460 (−0.849, −0.071)	**0.021**	**0.019**	−0.246 (−1.018, 0.526)	0.533	0.518
	*PTPRR* rs11178469	−0.127 (−0.415, 0.160)	0.386	0.384	−0.174 (−0.582, 0.234)	0.404	0.400	−0.144 (−0.685, 0.397)	0.602	0.600
**Δ LT**, mm/y	*PDGFRA* rs6554163	−0.029 (−0.071, 0.014)	0.190	0.187	−0.053 (−0.101, −0.004)	**0.033**	**0.033**	0.127 (−0.009, 0.263)	0.069	0.075
	*RASGRF1* rs6495367	−0.004 (−0.037, 0.028)	0.795	0.797	−0.046 (−0.101, 0.010)	0.106	0.109	0.029 (−0.023, 0.081)	0.272	0.280
	*PTPN5* rs155087*0*	0.040 (0.001, 0.079)	**0.043**	**0.041**	0.046 (−0.001, 0.093)	0.057	0.056	0.054 (−0.039, 0.148)	0.255	0.255
	*PTPRR* rs11178469	−0.031 (−0.066, 0.004)	0.084	0.080	−0.048 (−0.097, 0.001)	0.056	0.054	−0.024 (−0.090, 0.042)	0.478	0.484
**Δ ACD**, mm/y	*PDGFRA* rs6554163	−0.014 (−0.033, 0.006)	0.167	0.175	−0.009 (−0.031, 0.012)	0.404	0.411	−0.065 (−0.126, −0.004)	**0.038**	0.091
	*RASGRF1* rs6495367	0.011 (−0.004, 0.025)	0.162	0.170	0.006 (−0.019, 0.031)	0.638	0.647	0.021 (−0.002, 0.045)	0.076	0.066
	*PTPN5* rs155087*0*	0.000 (−0.017, 0.018)	0.966	0.967	−0.006 (−0.027, 0.016)	0.608	0.627	0.024 (−0.018, 0.066)	0.266	0.243
	*PTPRR* rs11178469	−0.006 (−0.022, 0.010)	0.452	0.454	−0.003 (−0.026, 0.019)	0.772	0.786	−0.016 (−0.045, 0.014)	0.303	0.256

*β^*a*^ is the coefficient of linear regression indicating the sizes of effects on changes in SE, AL, CRC, CCT, LT, and ACD during the 3.5-year follow-up. P^*a*^, adjusted for age, sex, near-work time, outdoor time, and the baseline of each outcome indicator (baseline SE for ΔSE, baseline AL for ΔAL, baseline CRC for ΔCRC, baseline CCT for ΔCCT, baseline LT for ΔLT and baseline ACD for ΔACD). P^*b*^, adjusted for age, sex, near-work time, outdoor time, and baseline of each outcome indicator with 10,000 permutations. The bold values are P < 0.05.*

All the results above remained consistent over 10,000 permutations, except for the relationship between *PDGFRA* rs6554163 and increased ACD (recessive: coefficient = −0.065, *p* = 0.038), which was no longer significant after the permutation test was conducted (adjusted *p* = 0.091).

### Gene-Gene Interaction Analysis

**FIGURE 1:**
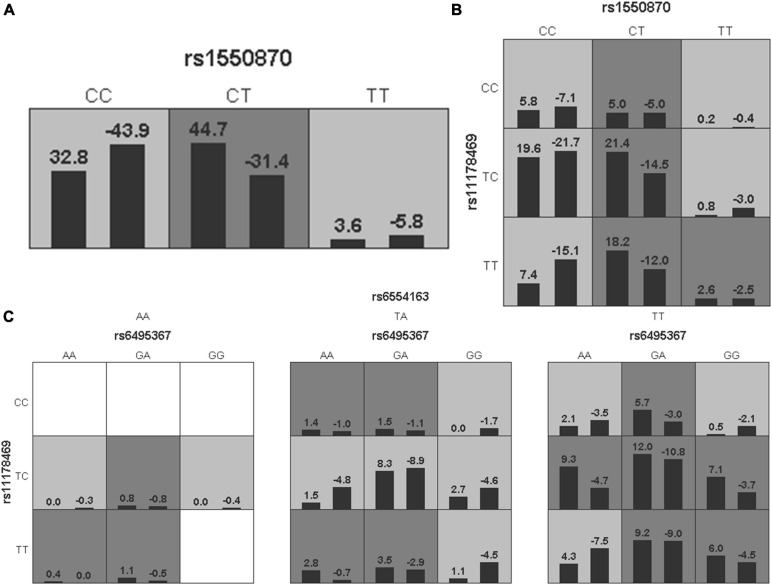
The three best models for predicting incident myopia given by GMDR analysis. **(A)** One-locus model of *PTPN5* rs1550870. **(B)** Two-loci model of *PTPN5* rs1550870-*PTPRR* rs11178469. **(C)** Three-loci model of *RASGRF1* rs6459367-*PTPRR* rs11178469-*PDGFRA* rs6554163. A grid represents the specific combinations of SNP-SNP interactions. High-risk genotypes are shown in dark gray, while low-risk genotypes are shown in light gray. All the bars on the left of each grid represent children who were not myopic at baseline but developed myopia in the follow-up period, while the bars on the right represent children who did not develop myopia during the complete follow-up period.

**FIGURE 2:**
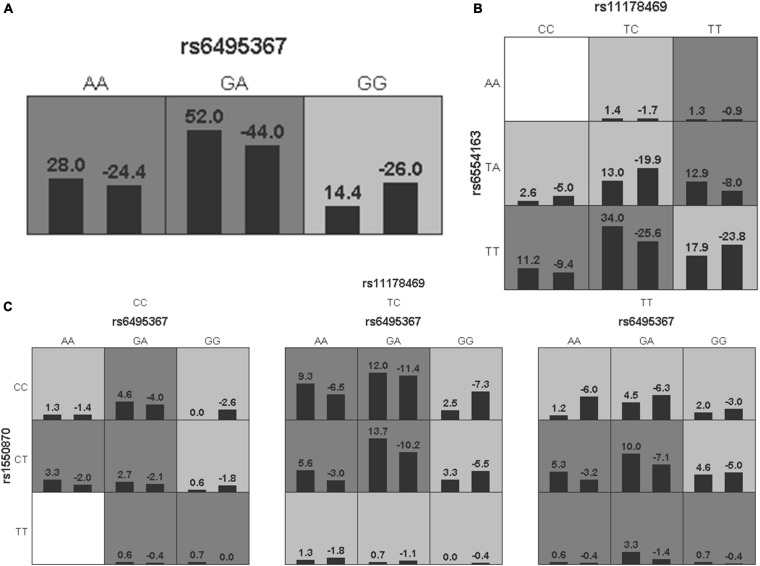
The three best models for predicting significant myopic shift given by GMDR analysis. **(A)** One-locus model of *RASGRF1* rs6495367. **(B)** Two-loci model of *PTPRR* rs11178469-*PDGFRA* rs6554163. **(C)** Three-loci model of *RASGRF1* rs6459367-*PTPN5* rs1550870-*PTPRR* rs11178469. A grid represents the specific combinations of SNP-SNP interactions. High-risk genotypes are shown in dark gray, while low-risk genotypes are shown in light gray. All the bars on the left of each grid represent cases, while the bars on the right represent controls.

To further analyze the impacts of the genetic interactions on myopia, especially incident myopia and significant myopic shift, GMDR was employed for logistic regression. As shown in Table 7 and Figure 1, evaluation of the effects of SNP-SNP combinations on incident myopia revealed a statistically significant one-locus model: *PTPN5* rs1550870 [cross-validation consistency (CVC) = 10/10, *p* = 0.0107]. With regard to the effects of the SNP-SNP interactions on significant myopic shift, the two-loci model consisting of *PTPRR* rs11178469 and *PDGFRA* rs6554163 had a significant *p*-value (0.0107) (Table 8 and Figure 2). However, the low cross-validation consistency (6/10) indicated an uncertain association of the combination with myopia.

**TABLE 7:** GMDR results of SNP-SNP interactions related to incident myopia.

Model	Training balanced accuracy	Testing balanced accuracy	Cross-validation consistency	Sign test (p)^*a*^
*PTPN5* rs155087*0*	0.5820	0.5751	10/10	**9 (0.0107)**
*PTPN5* rs155087*0*, *PTPRR* rs11178469	0.5871	0.5047	6/10	6 (0.3770)
*RASGRF1* rs6495367, *PTPRR* rs11178469, *PDGFRA* rs6554163	0.6183	0.4598	4/10	3 (0.9453)

*^*a*^Adjusted for sex, age, near-work time, outdoor time, and baseline SE. The bold values are P < 0.05.*

**TABLE 8:** GMDR results of SNP-SNP interactions related to significant myopic shift.

Model	Training balanced accuracy	Testing balanced accuracy	Cross-validation consistency	Sign test (p)^*a*^
*RASGRF1* rs6495367	0.5643	0.5324	7/10	7 (0.1719)
*PTPRR* rs11178469, *PDGFRA* rs6554163	0.5856	0.5346	6/10	**9 (0.0107)**
*RASGRF1* rs6495367, *PTPN5* rs155087*0, PTPRR* rs11178469	0.6144	0.5049	5/10	6 (0.3770)

*^*a*^Adjusted for sex, age, near-work time, outdoor time, and baseline SE. The bold values are P < 0.05.*

All the models used for GMDR linear regression on SE yielded non-significant results (*p* > 0.05, Supplementary Table 1 and Supplementary Figure 1).

### PPI Network

**FIGURE 3:**
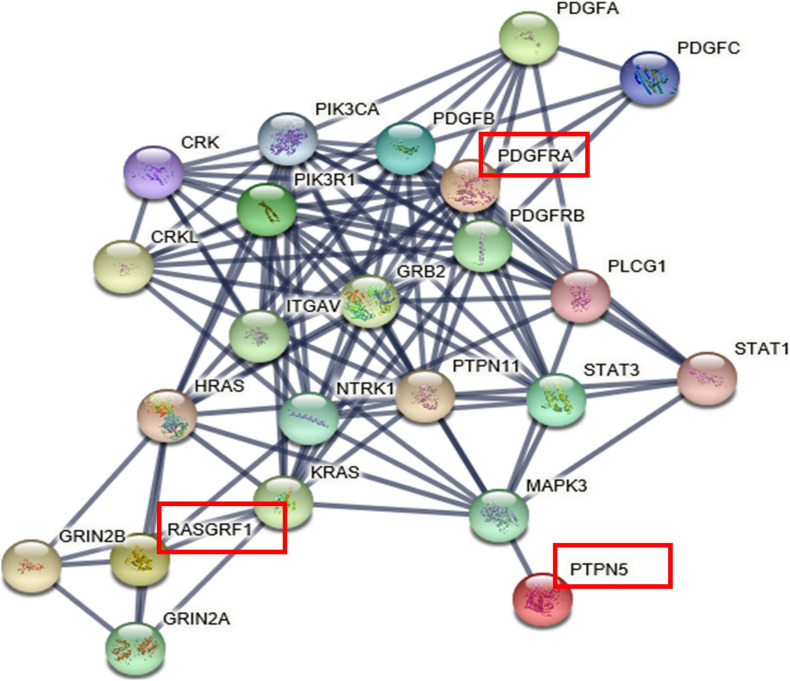
The protein-protein interaction network. The genes with red squares (including *PTPN5*, *RASGRF1*, and *PDGFRA*) were input into String.

The interaction network revealed direct and indirect partners of *RASGRF1*, *PTPN5*, and *PDGFRA* (Figure 3), and pathway enrichment analyses were performed on the set of genes in the network through String (Supplementary Table 1). The PPI network was constructed and was found to have 23 nodes connected by 114 edges. Each node represents a protein, and the edges represent the interactions between proteins. We observed that the three genes did not have direct interactions, but there were indirect connections through their intermediaries.

Pathway enrichment analysis also revealed that these genes regulate visual processes, including eye development, retinal vasculature development in camera-type eyes, visual learning, circadian entrainment, the light stimulus response, and dopaminergic synapse-related processes (all FDRs < 0.05).

### miRNA-Gene Regulatory Network

**FIGURE 4:**
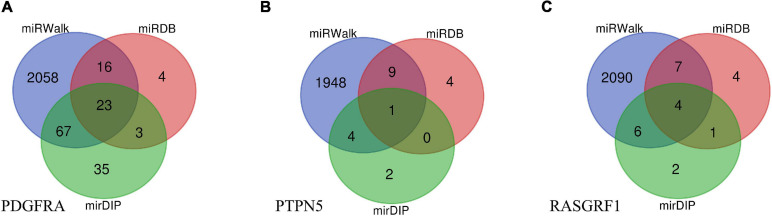
Venn diagrams of target miRNAs in different databases including miRWalk, miRDB, and mirDIP. **(A)** The miRNA distribution of *PDGFRA* in each database. **(B)** The miRNA distribution of *PTPN5* in each database. **(C)** The miRNA distribution of *RASGRF1* in each database.

Data from miRWalk, miRDB, and mirDIP predicted that a total of 28 miRNAs interact with the 3 genes (*PDGFRA*, *RASGRF1*, *PTPN5*). Venn diagrams were created to demonstrate the miRNA distribution in each database (Figure 4). Overall, 2,032 genes were determined by the three databases to be the target genes of these miRNAs.

**FIGURE 5:**
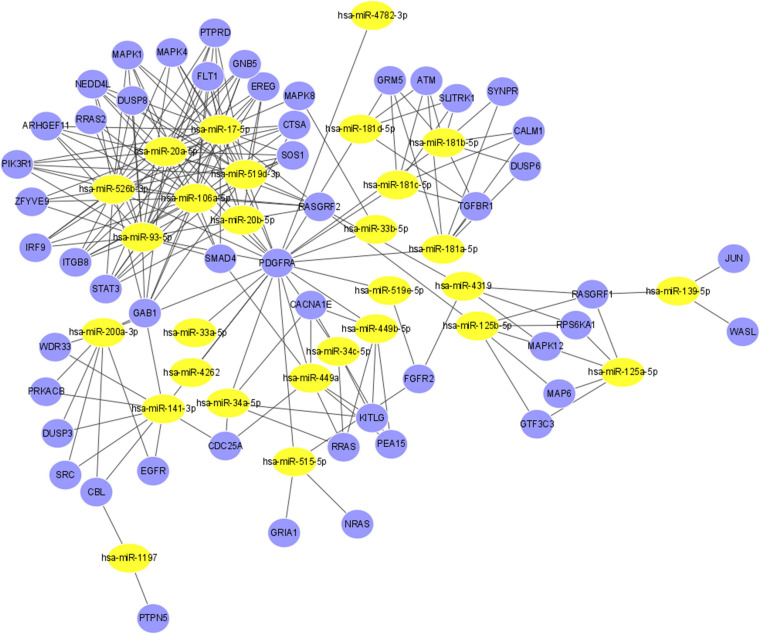
The miRNA-gene regulatory network. The yellow nodes represent 28 miRNAs, and the blue nodes represent the 51 genes that have direct or indirect interactions with *PDGFRA*, *RASGRF1*, and *PTPN5*. The edges represent the interaction relationships between miRNAs and genes.

String was then used to screen more relevant genes. Based on the intersection of the data obtained from String and the three databases, the regulatory functions of interactions among 28 miRNAs and 51 genes were investigated (Supplementary Table 2). Figure 5 shows the final miRNA-gene network, which consisted of 79 nodes and 211 edges.

KEGG and GO analyses of the 51 genes suggested that they are involved in eyelid development in camera-type eyes, Ras protein signal transduction, regulation of the ERK1 and ERK2 cascades, eye development, camera-type eye development, Ras guanyl-nucleotide exchange factor activity and dopaminergic synapse-related processes (Supplementary Table 2, all FDRs < 0.01).

## Discussion

Numerous researchers have concluded from mammalian and non-mammalian model studies that DA plays a critical role in the development of myopia (Zhou et al., 2017). The ERK signaling pathway is one of the downstream cascades of DA receptors expressed in retinal ganglia and photoreceptors. In this study, the associations of four selected gene variants in the ERK pathway with myopia and ocular parameters were assessed through longitudinal data obtained from Chinese primary school students to explore the role of the ERK pathway in human myopia. In the present study, *PTPN5* rs1550870 was found to be correlated with incident myopia, significant myopic shift and increasing LT as well as AL, but it had a negative effect on CCT. *RASGRF1* rs6495367 was negatively associated with myopic shift as well as myopic SE and AL. *PDGFRA* rs6554163 was negatively associated with increasing LT. Based on the results from GMDR, the one-locus model PTPN5 rs1550870 was significantly associated with incident myopia, which was consistent with the results of the single-locus analysis. Enrichment analyses of the genes in the PPI and miRNA-gene interaction networks suggested that these genes are involved mainly in eye development and dopaminergic synapse-related processes.

In the ERK signaling pathway, the autophosphorylation of receptor tyrosine kinase (RTK), which is activated by growth factor (GF) binding, generates binding sites to make GF receptor-bound protein 2 (GRB2) dock with Ras/Rac guanine nucleotide exchange factor 1 (SOS) and promotes the activation of these two molecular complexes (Mendoza et al., 2011). PDGF receptor (PDGFR) is a member of the RTK family (Velghe et al., 2014). SOS catalyzes Ras GTP, and activated Ras-GTP then recruits Raf to the membrane, where it is activated (Mendoza et al., 2011). RASGRF1 is one of the nucleotide exchange factors that activates Ras (Barman et al., 2014). Raf, as a Ras effector, activates MAPK ERK kinase (MEK) through double phosphorylation on serine residues after binding Ras (Zhang and Guan, 2000). ERK is a core MAPK component that functions as the major effector of the Ras protein (Mendoza et al., 2011). Striatal-enriched tyrosine protein phosphatase (STEP), encoded by the *PTPN5* gene, can limit ERK activity as well as subsequent downstream nuclear signaling (Paul et al., 2003). In addition, an important paralog of the *PTPN5* gene is *PTPRR*, which is a key negative regulator of ERK (Shi et al., 2012).

The *PDGFRA* gene binds three forms of *PDGF* (PDGF-AA, PDGF-AB, and PDGF-BB) and mediates many biological processes (Sulzbacher et al., 2008). Previous studies on humans as well as animals have demonstrated that the expression of *PDGF-AA* in the retinal pigment epithelium (RPE) is increased under pathological conditions (Andrews et al., 1999). In a recent study combining two meta-analyses from CREAM and UK Biobank, *PDGFRA* was found to be strongly associated with corneal curvature (*P* = 1.59 × 10^–73^) (Fan et al., 2020). In the present study, *PDGFRA* rs6554163 was discovered to be correlated with LT. The non-significant results regarding the association of *PDGFRA* rs6554163 with AL are similar to those from a study conducted in Asia but different from those from a study conducted in Europe (Guggenheim et al., 2013; Chen et al., 2014, 2017). One possible explanation for the variation might be ethnic differences.

To date, there have been many studies investigating the association between SNPs in *RASGRF1* and myopia. Our finding that *RASGRF1* rs6495367 is significantly related to hypermetropic SE is consistent with the findings of a previous GWAS meta-analysis involving 160,420 Asian and European individuals (Tedja et al., 2018). In another GWAS involving 3,269 Japanese participants, the association between *RASGRF1* and myopia was confirmed (Meguro et al., 2020). Recent studies have hypothesized that *RASGRF1* is linked to the dopaminergic system (Gong et al., 2017). Photoreceptors can detect light intensity and image contrast, both of which are capable of regulating the amount of DA and thus influencing myopia (Zhou et al., 2017). In addition, one study emphasized the role of *RASGRF1* as an exchange factor in the synaptic transmission of photoreceptor responses (Fernández-Medarde et al., 2009). Thus far, there have been few studies on the association between *RASGRF1* SNPs and AL. However, in our research, *RASGRF1* rs6495367 was found to be associated with AL. It has been reported that *RASGRF1* protein expression can be altered by changes in retinoic acid and muscarinic receptor levels (Mattingly and Macara, 1996; Tideman et al., 2018). In this respect, the eye may respond to retinoic acid and adjust its axial elongation (McFadden et al., 2004). Therefore, the association of *RASGRF1* rs6495367 with SE and AL may be interpreted as a joint effect of DA and retinoic acid.

*PTPN5* rs155087*0* was found to be strongly associated with myopia only in a large-sample GWAS based on a European population (*P* = 9.9 × 10^–13^) (Pickrell et al., 2016). Neither myopia nor ocular parameters have been reported to be related to SNPs among Asian populations. In the current study, *PTPN5* rs155087*0* was significantly associated with incident myopia and significant myopic shift. *PTPN5* (also named striatal-enriched *PTP*, *STEP*) tends to be expressed in neurons of the central nervous system, where it regulates the neurotransmission of DA (Eswaran et al., 2006). Accumulating evidence is emerging for the important roles of diurnal and circadian rhythms in eye growth and refractive error development, in which intrinsically photosensitive RGCs (ipRGCs) govern visual input. The functions of DA in the mechanism above include not only the simple alignment of intrinsic retinal rhythms to the light-dark cycle but also the adjustment of refractive development (Chakraborty et al., 2018). DA may exert its effects through RGCs and regulate the surroundings of the RGC receptive field. The MAPK/ERK signaling pathway may also be involved in the above modulation of neuronal functions (Li et al., 2016). Given that PTPN5 serves as a downstream component that adjusts the duration and functions of ERK signaling and that it is expressed specifically in rat RGCs (Paul et al., 2003; Li et al., 2016), we hypothesize that the relationship of *PTPN5* with myopia may be related to the role of DA in refractive error. Several reports have shown that as a retinal signal, dopamine can regulate eye growth through remodeling of the scleral extracellular matrix (ECM) (Wojciechowski and Hysi, 2013). Moreover, abnormal scleral ECM remodeling and the concomitant excess elongation of axial length can lead to myopia, which involves gene-expression changes associated with the phenotypic transdifferentiation of Fib-L toward Myofib-L. *PTPN5* is one of the differentially expressed genes in this process (Wu et al., 2018). In our study, *PTPN5* rs1550870 was associated with AL, further supporting our hypothesis stated above. In addition, *PTPN5* rs155087*0* was linearly correlated with LT and CCT in the current study. However, its specific mechanisms related to LT and CCT remain to be clarified.

Within the human eye, the increased expression of *PTPRR* has been previously reported in rapidly growing fetal retina/RPE tissue (*PTPRR* expression has not been detected in adult RPE tissue) and choroid tissue, suggesting that PTPRR controls ocular growth. In a Caucasian family cohort study, *PTPRR* rs3803036 was found to be strongly associated with high myopia (Hawthorne et al., 2013). Meta-analyses of the genome-wide single variant *PTPRR* rs11178469 have shown a linear relationship between this variant and refractive error in mixed ancestries, including Asian and European ancestries (Tedja et al., 2018). However, in our single-locus analyses, we did not find any evidence to support the association of myopia or ocular parameters with *PTPRR* rs11178469. This result is similar to the results of a study by Yoshikawa et al. (2014). Explorations with large cohorts should be conducted in different ethnic groups to further evaluate the role of *PTPRR* and the impacts of *PTPRR* on myopia risk factors.

Our functional prediction indicated that both *PDGFRA* rs6554163 and *RASGRF1* rs6495367 can change the SIX5 motif. It has been reported that SIX5 transcripts are detectable in the epithelium of the adult cornea and lens as well as in the cellular layers of the retina and sclera (Winchester et al., 1999). Therefore, these two SNPs may affect myopia by changing the SIX5 motif. *PTPN5* rs155087*0* alters motifs of the Krüppel-like factor family, which are enriched in corneal epithelial enhancers. Furthermore, KLF7 acts as an antagonist of KLF4 in the differentiation of corneal epithelial cells (Klein et al., 2017). A disruption of the balance between the levels of these two factors may be related to the expression of *PTPN5* rs155087*0* and, in turn, influence the eye to some extent.

GMDR analyses demonstrated a significant correlation between the one-locus model (*PTPN5* rs1550870) and incident myopia. We can therefore infer that *PTPN5* rs1550870 may be an independent risk factor for this disease. Additionally, in the logistic regression analysis of significant myopic shift, the combination of *PTPRR* rs11178469 and *PDGFRA* rs6554163 had a significant *p*-value and a low CVC (6/10). The result therefore needs to be interpreted with caution and to be confirmed in studies on larger sample sizes and additional ethnic groups.

Enrichment analyses showed that genes utilized in the construction of the PPI and miRNA-gene interaction networks may regulate biological processes such as retinal vasculature development in camera-type eyes, the response to light stimulus, dopaminergic synapse-related processes and the ERK1/ERK2 cascade. Of note, a review summarized the locations of DA receptors, including RGCs, RPE cells and photoreceptors, in mammalian retinas (Nguyen-Legros et al., 1999). Therefore, we suggest that the regulation of myopia by DA may be related to the ERK pathway. Given the specific expression of these three genes at these locations and the function of DA in myopia, the findings may provide further insight into the biological mechanisms by which DA regulates myopia progression.

Large-scale studies have indicated the pivotal roles of miRNAs in the development of myopia (Tkatchenko et al., 2016). In the current study, we created a network including 28 miRNAs and 51 genes. Hsa-miR-17-5p, one of the miRNAs with the most gene interactions, was identified as a myopia-specific miRNA in a previous study (Chen et al., 2019). Changes in the expression of miRNAs can influence a whole genetic network and alter the corresponding phenotype via PPIs. By revealing the potential relationships between genes and miRNAs correlated with *PDGFRA*, *RASGRF1*, and *PTPN5*, this study may shed new light on myopia at the molecular level.

To our knowledge, this is the first study on the association of *PTPN5* rs1550870 with myopia via GWAS. We also evaluated the roles of *RASGRF1* rs6495367, *PDGFRA* rs6554163, and *PTPRR* rs11178469 in myopia in a southern Chinese Han population.

In the current analysis, in addition to the SE, other ocular parameters related to myopia were included as outcome indicators. Previous studies have revealed that the corneal system, lens system, ACD, and AL represent the refractive components that determine the refractive state. Flattening of the cornea and lens alleviates the influence of axial elongation on the refractive state (Mutti et al., 2005). Four variants of four genes from the ERK pathway were chosen for analysis, three of which were proven to be associated with myopia or altered ocular parameters. The ERK signaling pathway is known as one of the downstream signaling cascades of DA. Moreover, bioinformatic analyses uncovered the involvement of relevant genes and miRNAs in ocular development and revealed the role of DA in biological functional regulation. The above information can be effectively combined to provide quantitative insights into the role of the ERK signaling pathway in the mechanism by which DA inhibits myopia; the results suggest that this pathway may be the downstream signaling pathway of DA receptors in the retina. In this 3.5-year longitudinal study, we calculated the annual average variation in each ocular parameter to explore the association of the parameters with genetic variants, and the evidence supports the idea that these SNPs are likely to impact the dynamic process of myopia.

However, there were some limitations of this study. First, the SE data were gathered using a non-cycloplegic autorefraction assessment. We cannot exclude the possibility that this method may have failed to reflect subjects’ actual levels of refractive error and therefore overestimated myopia. To reduce the overestimation of myopia levels, we chose a SE ≤ −1.0 D as the definition of myopia. Second, the statistical performance of our study might have been restricted by the small sample size. Research on a larger population is warranted to confirm the results. Third, we chose only a single SNP of each gene for genotyping, which may have caused some information to be lost due to the insufficient coverage of variants, highlighting the need for future studies to comprehensively examine the correlation of more variants with myopia. In addition, it is noteworthy that permutation test in our study may has a deficiency, for we also tested for the relationships between each marker with multiple traits under different models. Therefore, caution is needed when interpreting the results. At last, the specific mechanisms remain unknown. Therefore, experimental animal models should be used in future research to determine the potential corresponding mechanisms.

## Conclusion

Through a longitudinal study conducted on primary school students, we identified crucial genes in the ERK signaling pathway that are closely correlated with myopia. Our findings suggest that *PTPN5* rs1550870 and *RASGRF1* rs6495367 are associated with the susceptibility to myopia and changes in several ocular parameters in southern Chinese children. *PDGFRA* rs6554163 is related to LT. Therefore, the ERK signaling pathway may play a role in the DA-mediated control of myopia. Additionally, we combined gene and miRNA functional analyses with GO and KEGG analyses to emphasize the regulatory effects associated with ocular development and DA biological functions. The results reported in this study can offer novel clues for screening and understanding the molecular mechanisms underlying the pathogenesis of myopia. However, further molecular biological studies are required to verify these findings.

## Data Availability Statement

The data in this study are available from the corresponding author YC, wzcyymail@163.com, upon reasonable request.

## Ethics Statement

The studies involving human participants were reviewed and approved by the Ethics Committee of the Eye Hospital of Wenzhou Medical University. Written informed consent to participate in this study was provided by the participants’ legal guardian/next of kin.

## Author Contributions

YC contributed conception and design of the study. DJ, YL, LL, QZ, and JH organized the database. HX and SL performed the statistical analysis and wrote the manuscript. All authors contributed to the manuscript revision and read and approved the submitted version.

## Conflict of Interest

The authors declare that the research was conducted in the absence of any commercial or financial relationships that could be construed as a potential conflict of interest.

## Footnotes

1 https://www.string-db.org/

2 http://mirwalk.umm.uni-heidelberg.de/

3 http://mirdb.org/

4 http://ophid.utoronto.ca/mirDIP/

5 http://bioinformatics.psb.ugent.be/webtools/Venn/

6 https://cytoscape.org/index.html
