# Supplementary material for: Association of Extracellular Signal-Regulated Kinase Genes With Myopia: A Longitudinal Study of Chinese Children
Source: Front Genet. 2021 May 27;12:654869. doi: 10.3389/fgene.2021.654869 (PMC8191505; doi:10.3389/fgene.2021.654869)
Supplement: Supplementary file 1 [file Data_Sheet_1.docx]

***Supplementary Material***

**Supplementary Table 1.** GMDR results of SNP-SNP interaction related to the change of SE.

| **Model** | **Training balanced accuracy** | **Testing balanced accuracy** | **Cross-validation consistency** | **Sign test (p)^a^** |
| --- | --- | --- | --- | --- |
| *RASGRF1* rs6495367 | 0.5754 | 0.5347 | 8/10 | 8 (0.0547) |
| *RASGRF1* rs6495367, *PTPN5* rs1550870 | 0.6054 | 0.5559 | 5/10 | 7 (0.1719) |
| *RASGRF1* rs6495367, *PTPRR* rs11178469, *PDGFRA* rs6554163 | 0.6339 | 0.5526 | 5/10 | 8 (0.0547) |

^a^, adjusted for gender, age, near-work time, outdoor time, and baseline SE.

**Supplementary Table 2.** Target miRNAs and genes from the intersection of String and three databases.

| **MicroRNA** | **Gene** |  | |  |  |
| --- | --- | --- | --- | --- | --- |
| hsa-miR-17-5p | PIK3R1 |  | | |  |
| hsa-miR-106a-5p | STAT3 |  |  |  |  |
| hsa-miR-526b-3p | WDR33 |  |  |  |  |
| hsa-miR-20a-5p | CTSA |  |  |  |  |
| hsa-miR-93-5p | DUSP3 |  |  |  |  |
| hsa-miR-519d-3p | DUSP6 |  |  |  |  |
| hsa-miR-20b-5p | DUSP8 |  |  |  |  |
| hsa-miR-141-3p | GRIA1 |  |  |  |  |
| hsa-miR-200a-3p | GRM5 |  |  |  |  |
| hsa-miR-181a-5p | GTF3C3 |  |  |  |  |
| hsa-miR-181b-5p | JUN |  |  |  |  |
| hsa-miR-515-5p | MAP6 |  |  |  |  |
| hsa-miR-181c-5p | MAPK1 |  |  |  |  |
| hsa-miR-181d-5p | MAPK12 |  |  |  |  |
| hsa-miR-125a-5p | MAPK4 |  |  |  |  |
| hsa-miR-125b-5p | MAPK8 |  |  |  |  |
| hsa-miR-139-5p | NEDD4L |  |  |  |  |
| hsa-miR-4319 | PEA15 |  |  |  |  |
| hsa-miR-33b-5p | PTPN5 |  |  |  |  |
| hsa-miR-34c-5p | PTPRD |  |  |  |  |
| hsa-miR-449a | RPS6KA1 |  |  |  |  |
| hsa-miR-449b-5p | SMAD4 |  |  |  |  |
| hsa-miR-1197 | TGFBR1 |  |  |  |  |
| hsa-miR-34a-5p | SYNPR |  |  |  |  |
| hsa-miR-519e-5p | SLITRK1 |  |  |  |  |
| hsa-miR-4262 | ZFYVE9 |  |  |  |  |
| hsa-miR-4782-3p | ARHGEF11 |  |  |  |  |
| hsa-miR-33a-5p | ATM,CACNA1E, CALM1, CDC25A, GNB5, NRAS, PRKACB, RASGRF1, RASGRF2, RRAS, RRAS2, SOS1, SRC, WASL, CBL, EGFR, EREG, FGFR2, FLT1, GAB1, IRF9, ITGB8, KITLG, PDGFRA | |  | | |

**
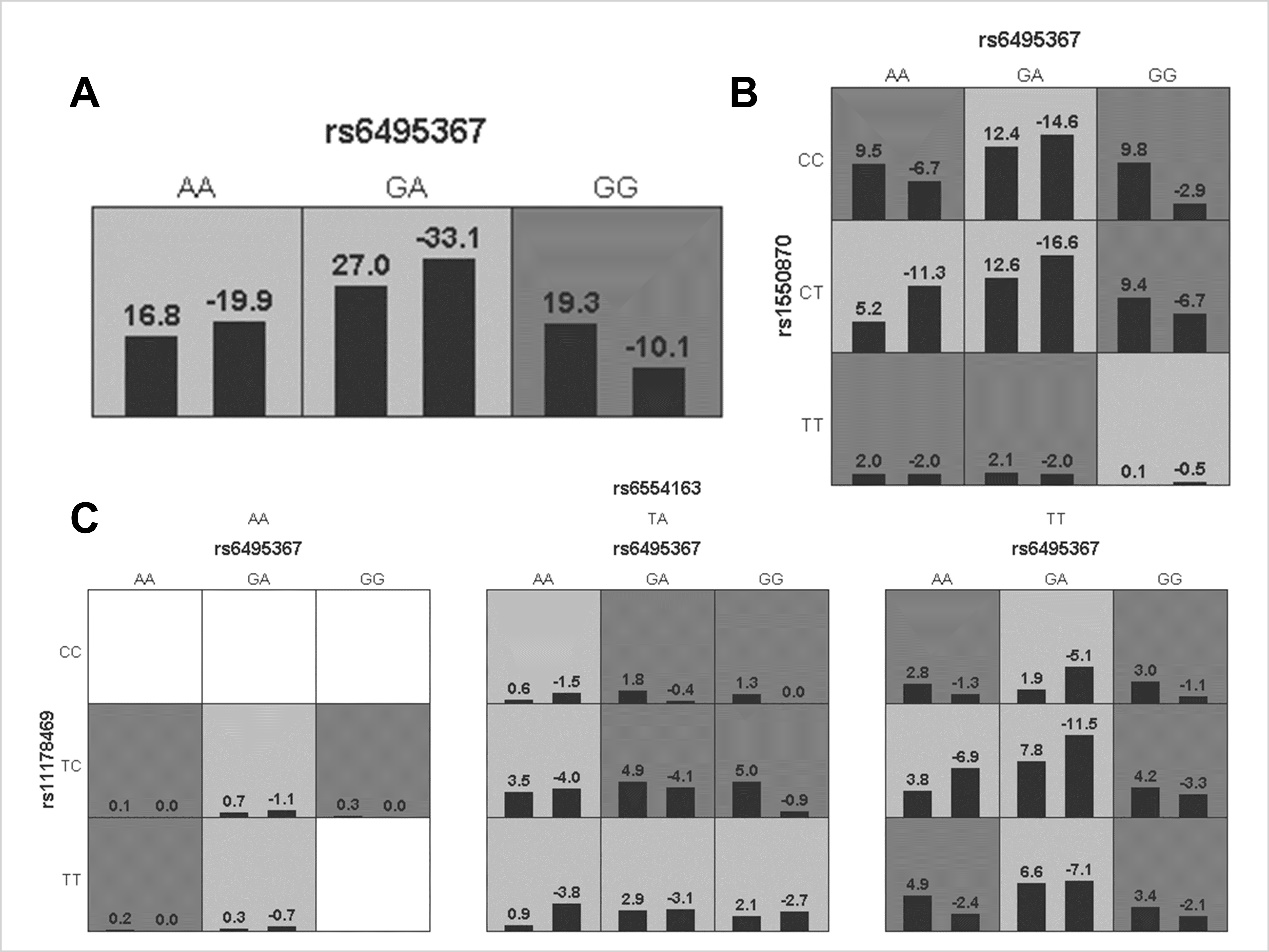
**

**Supplementary Figure 1.** The three best models for predicting the change of SE are given by GMDR analysis. **(A)** One-locus model of *RASGRF1* rs6495367. **(B)** Two-loci model of *RASGRF1* rs6495367-*PTPN5* rs155087*0*. **(C)** Three-loci model of *RASGRF1* rs6459367-*PTPRR* rs11178469-*PDGFRA* rs6554163. A grid represents the specific combination of SNP-SNP interaction. High-risk genotypes are shown in dark gray, while low-risk genotypes are shown in light gray. All the left bars of each grid represent cases, while the bars on the right represent controls.
